# Protective action of natural and induced immunization against the occurrence of delta or alpha variants of SARS-CoV-2 infection: a test-negative case-control study

**DOI:** 10.1186/s12916-022-02262-y

**Published:** 2022-02-08

**Authors:** Giovanni Corrao, Matteo Franchi, Federico Rea, Danilo Cereda, Antonio Barone, Catia Rosanna Borriello, Petra Giulia Della Valle, Michele Ercolanoni, Ida Fortino, Jose Jara, Olivia Leoni, Francesco Mazziotta, Elisabetta Pierini, Giuseppe Preziosi, Marcello Tirani, Massimo Galli, Guido Bertolaso, Giovanni Pavesi, Francesco Bortolan

**Affiliations:** 1 National Centre for Healthcare Research and Pharmacoepidemiology, University of Milano-Bicocca, Milan, Italy; 2 Unit of Biostatistics, Epidemiology and Public Health, Department of Statistics and Quantitative Methods, University of Milano-Bicocca, Milan, Italy; 3 Directorate General for Health, Lombardy Region, Milan, Italy; 4 ARIA S.p.a., Milan, Italy; 5 Infectious Diseases Unit, Luigi Sacco Hospital, Milan, Italy; 6 Department of Biomedical and Clinical Sciences, University of Milan, Milan, Italy; 7 Chief of the Regional staff for the management of the vaccination campaign, Lombardy Region, Milan, Italy

**Keywords:** SARS-CoV-2, SARS-CoV-2 variants, Vaccination, Immunization, Public health

## Abstract

**Background:**

The evolution of SARS-CoV-2 has led to the emergence of several new variants, and few data are available on the impact of vaccination on SARS-CoV-2 variants. We aimed to assess the association between natural (previous infection) and induced (partial or complete vaccination) exposure to SARS-CoV-2 and the onset of new infection supported by the delta variant, and of comparing it with that supported by alpha.

**Methods:**

We performed a test-negative case-control study, by linking population-based registries of confirmed diagnoses of infection with SARS-CoV-2, vaccinations against Covid-19 and healthcare utilization databases of the Italian Lombardy Region. Four hundred ninety-six persons who between 27 December 2020 and 16 July 2021 had an infection by the delta variant were 1:1 matched with citizens affected by alphavariant and 1:10 matched with persons who had a negative molecular test, according to gender, age and date of molecular ascertainment. We used a conditional logistic regression for estimating relative risk reduction of either variants associated with natural and/or induced immunization and corresponding 95% confidence interval (CI).

**Results:**

Previous infection was associated with 91% (95% CI 85% to 95%) reduced relative risk of reinfection, without evidence of significant differences between delta and alpha cases (*p*=0.547). Significant lower vaccinal protection against delta than alpha variant infection was observed with reduced relative risk associated with partial vaccination respectively of 29% (7% to 45%), and 62% (48% to 71%) (*p*=0.001), and with complete vaccination respectively of 75% (66% to 82%) and 90% (85% to 94%) (*p*=0.003).

**Conclusions:**

Lower protection towards infections caused by the delta variant with respect to alpha variant was noticed, even after the completion of the vaccination cycle. This finding would support efforts to maximize both vaccine uptake with two doses and fulfilment with individual protection measures, especially as the delta variant is rampant worldwide presently.

**Supplementary Information:**

The online version contains supplementary material available at 10.1186/s12916-022-02262-y.

## Background

The evolution of severe acute respiratory syndrome-coronavirus (SARS-CoV)-2 has led to the emergence of several new variants. Among these, “Variants of Concern” (VoC) are defined by phenotypic changes, including enhanced transmission, increased pathogenicity and reduced efficacy of prophylactic and therapeutic countermeasures [1]. Based on an epidemiological update from the World Health Organization on 22 June 2021, four VoC have been identified since the beginning of the pandemic: alpha (B.1.1.7; this was the first VoC described in the UK in late-December 2020), beta (B.1.351; South Africa, December 2020), gamma (P.1, Brazil; early-January 2021) and delta (B.1.617.2; India, December 2020) [2]. People infected with the delta variant have been described as having a viral load ~1000-times higher than that in people infected with the original SARS-CoV-2 [3], so the delta variant spreads faster than other variants [4].

VoC display specific mutations in the spike protein that reduce the binding affinity of neutralising antibodies [5–7]. The beta variant, gamma variant, and delta variant have shown significant escape from natural infection-mediated neutralisation [8]. Levels of neutralising antibodies induced by m-RNA vaccines against SARS-CoV-2 variants have been found to be similar (or higher) than those derived from naturally infected individuals [9]. Although modest differences in vaccine efficacy were noted recently with the delta variant as compared with the alpha variant after receipt of two vaccine doses [10], too few and inconsistent data hamper addressing of vaccine policies while variants are spreading.

We undertook a case-control investigation during the ongoing vaccination campaign in the Italian region of Lombardy. We aimed to estimate the association between natural (ascertained previous infection) and/or induced (partial or complete administration of a vaccine) exposure to SARS-CoV-2, and the onset of new infection due to the delta variant with respect to the alpha variant (which are the variants covering ~90% of SARS-CoV-2 infections occurring currently in the study population).

## Results

**Fig. 1:**
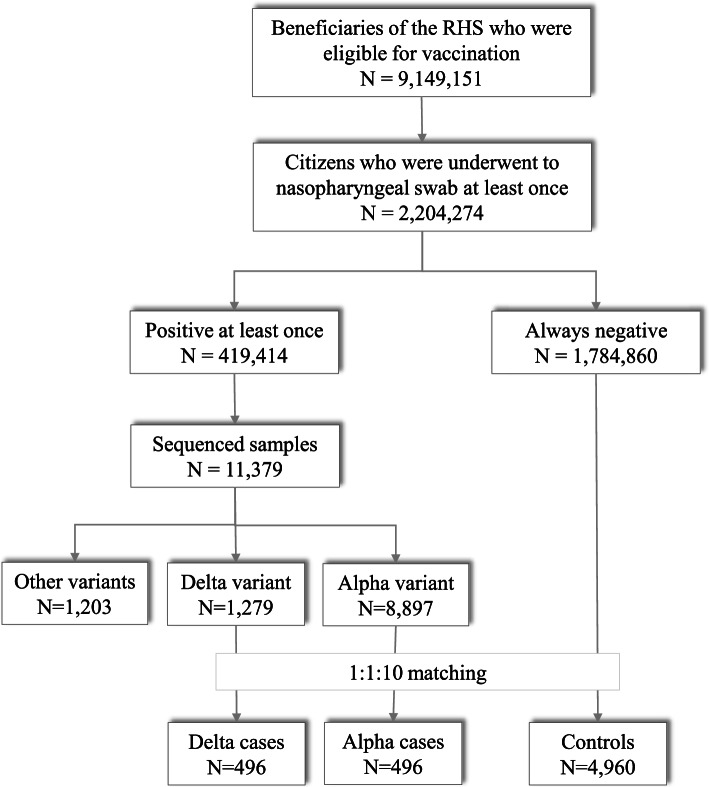
Selection process of study population starting from candidates to vaccination identified at December 27, 2020, until the case identification (i.e., individuals for whom an infection caused by delta or alpha variant was ascertained from April 3, 2021, until July 16, 2021), and their inclusion by means of a matching design jointly with negative controls

Just over 9 million beneficiaries of the RHS aged 12 years or older were candidates for vaccination against COVID-19 on 27 December 2020 (i.e., when the vaccination campaign started in Italy). During the vaccination campaign (from 27 December 2020 until 16 July 2021, i.e., the endpoint of the current study) just over 2-million candidates for vaccination had provided a nasopharyngeal swab for testing at least once. Among them, ~419,400 individuals (~19%) were positive at least once according to a molecular test. Whole-genome sequencing was used to identify VoC for 11,300 individuals (2.3% of positive samples). The proportion of samples that were sequenced increased from 0.8% in January 2021 to 26.9% in July 2021. During the study period, 1279 and 8897 sequenced samples tested positive for the delta variant and alpha variant, respectively. Among these, 496 delta cases were matched to 496 alpha cases and to 4960 controls (Fig. 1). Interestingly, a delta variant was first found on 3 May 2021 and, since then, the proportion of delta variants went from 0.9% in May to 62.4% in the first half of July 2021. Conversely, the proportion of the alpha variant continuously decreased from May to July 2021. This explains why, among the 1279 individuals found with the delta variant, only 496 were matched and included in the study. Additional File 1: Fig. S1 shows the trend of new infections sustained by delta and alpha variants observed during the study period. There was no substantial difference in baseline characteristics between the 496 delta patients included in the study and the 783 who did not match on alpha patients and were therefore excluded. Included and excluded delta patients, respectively, were 55% and 57% of male gender, 71% and 74% were aged 49 years or younger, 74% and 71% had few previous contacts with the NHS and 91% and 92% were asymptomatic or puacisymptomatic at the time of infection detection.

### Cases and controls

Compared with controls, people infected with either delta variant or alpha variant had less frequent (i) previous contact with the RHS, (ii) previous use of the drugs under consideration (mainly corticosteroids and oral anticoagulants) and (iii) current presence of several diseases among those considered (mainly anaemia, depression, epilepsy, psychosis, malignancies, other diseases of the respiratory and genitourinary systems, symptoms, signs and ill-defined conditions, and other psychiatric disorders) (Table 1). Conversely, there was no statistical evidence that delta variant-infected cases and alpha variant-infected patients differed in any of the characteristics under consideration, or for COVID-19 severity.

**Table 1:** Comparing matched delta and alpha cases and controls for selected characteristics

	Delta variant *N* = 496	Alpha variant *N* = 496	Controls *N* = 4960	*p* value ^†^		
				Delta vs. controls	Alpha vs. controls	Delta vs. Alpha
Men	274 (55.2%)	274 (55.2%)	2740 (55.2%)	Matching variable		
Age category						
12 to 49 years	354 (71.4%)	354 (71.4%)	3540 (71.4%)	Matching variable		
50 to 59 years	76 (15.3%)	76 (15.3%)	760 (15.3%)			
60 to 69 years	36 (7.3%)	36 (7.3%)	360 (7.3%)			
70 to 79 years	18 (3.6%)	18 (3.6%)	180 (3.6%)			
≥ 80 yr	12 (2.4%)	12 (2.4%)	120 (2.4%)			
Number of previous contacts with NHS ^a^						
< 5	317 (63.9%)	321 (64.7%)	2893 (58.3%)	0.0160	0.0058	0.7909
5 to 99	166 (33.5%)	159 (32.1%)	1775 (35.8%)			
≥ 100	13 (2.6%)	16 (3.2%)	292 (5.9%)			
Users of selected drugs ^a b^						
Corticosteroids	36 (7.3%)	44 (8.9%)	576 (11.6%)	0.0034	0.0666	0.3511
Drugs for chronic pain	5 (1.0%)	7 (1.4%)	142 (2.9%)	0.0150	0.0586	0.5614
Oral anticoagulant agents	2 (0.4%)	2 (0.4%)	98 (2.0%)	0.0128	0.0128	1.0000
Insulin	6 (1.2%)	8 (1.6%)	94 (1.9%)	0.2342	0.5766	0.5904
Patients suffering selected diseases ^a b^						
Anaemias	28 (5.6%)	31 (6.3%)	484 (9.8%)	0.0027	0.0108	0.6873
Chronic respiratory disease	39 (7.9%)	55 (11.1%)	453 (9.1%)	0.3464	0.1530	0.0831
Dyslipidaemia	30 (6.0%)	23 (4.6%)	404 (8.1%)	0.0999	0.0055	0.3233
Depression	24 (4.8%)	21 (4.2%)	385 (7.8%)	0.0184	0.0043	0.6473
Hypertension	29 (5.8%)	28 (5.6%)	362 (7.3%)	0.2321	0.1730	0.8916
Coronary and peripheral vascular disease	28 (5.6%)	23 (4.6%)	351 (7.1%)	0.2319	0.0404	0.4725
Hypothyroidism	12 (2.4%)	10 (2.0%)	190 (3.8%)	0.1125	0.0403	0.6664
Epilepsy and recurrent seizures	4 (0.8%)	5 (1.0%)	155 (3.1%)	0.0034	0.0077	0.7378
Psychosis	1 (0.2%)	6 (1.2%)	144 (2.9%)	0.0004	0.0279	0.0579
Diabetes without insulin therapy	17 (3.4%)	11 (2.2%)	142 (2.9%)	0.4761	0.4066	0.2503
Malignancies	4 (0.8%)	6 (1.2%)	139 (2.8%)	0.0080	0.0355	0.5251
Other diseases of the respiratory system	3 (0.6%)	6 (1.2%)	136 (2.7%)	0.0040	0.0410	0.3152
Other diseases of the digestive system	5 (1.0%)	13 (2.6%)	131 (2.6%)	0.0261	0.9787	0.0571
Other diseases of the genitourinary system	3 (0.6%)	3 (0.6%)	130 (2.6%)	0.0055	0.0055	1.0000
Gout	7 (1.4%)	9 (1.8%)	113 (2.3%)	0.2094	0.5054	0.6143
Autoimmune disease	5 (1.0%)	6 (1.2%)	109 (2.2%)	0.0774	0.1442	0.7618
Other diseases of the circulatory system	4 (0.8%)	3 (0.6%)	96 (1.9%)	0.0739	0.0343	0.7045
Symptoms, signs and ill-defined conditions	0 (0.0%)	2 (0.4%)	96 (1.9%)	0.0018	0.0143	0.1569
Diseases of the skin and subcutaneous tissues	4 (0.8%)	3 (0.6%)	82 (1.7%)	0.1488	0.0722	0.7045
Arrhythmia	4 (0.8%)	5 (1.0%)	74 (1.5%)	0.2201	0.3897	0.7378
Inflammatory bowel diseases	6 (1.2%)	5 (1.0%)	70 (1.4%)	0.7149	0.4621	0.7618
Other mental disorders	0 (0.0%)	0 (0.0%)	69 (1.4%)	0.0082	0.0082	-
Heart failure	2 (0.4%)	5 (1.0%)	64 (1.3%)	0.0849	0.5917	0.2553
Glaucoma	5 (1.0%)	6 (1.2%)	58 (1.2%)	0.7485	0.9366	0.7618
Chronic kidney disease	0 (0.0%)	2 (0.4%)	58 (1.2%)	0.0155	0.1188	0.1569
Severity of Covid-19 symptoms ^c^						
Asymptomatic	122 (24.6%)	122 (24.6%)	-	-	-	0.6414
Mild	330 (66.5%)	314 (63.3%)	-	-	-	
Severe	37 (7.5%)	51 (10.3%)	-	-	-	
Critical	4 (0.8%)	4 (0.8%)	-	-	-	
Fatal	3 (0.6%)	5 (1.0%)	-	-	-	

^†^ Chi-square test, or its version for the trend (categories of previous contacts with NHS and severity of Covid-19 symptoms)

^a^Measured in the 2-years period before the index date

^b^Items have been sorted by decreasing frequency among controls. With respect to the complete investigated lists (please see Additional file 1: Table S3), only drugs used and diseases suffered by at least 1.0% of controls (i.e., by at least 50 negative-test citizens among the 4,960 ones included into the study) are reported

^c^ Severity recognized at the index date. Source: https://www.COVID19treatmentguidelines.nih.gov/overview/clinical-presentation/#:~:text=Moderate%20COVID%2D19%20illness%20is,with%20moderate%20disease%20is%20recommended

### Effect of natural and induced exposure to SARS-CoV-2

Significant protective action of natural exposure against the occurrence of SARS-CoV-2 reinfection as a whole, as well as of symptomatic COVID-19 illness was observed, with relative risk reductions of 88% (79% to 93%) and 90% (81% to 95%), respectively. Among patients who experienced delta- and alpha-variant infection, six (1.2%) and 9 (1.8%) had previous natural exposure to SARS-CoV-2, with median (minimum-maximum) time of reinfection of 207 (115–529) days and 127 (94–453) days, respectively. Table 2 shows that between-variant differences in relative risk reductions for the association between natural exposure and both SARS-CoV-2 reinfection and symptomatic COVID-19 illness were weak and not significant. Partial vaccination and complete vaccination were associated with significant protection against SARS-CoV-2 infection as a whole, and even more towards symptomatic COVID-19 illness, being the corresponding relative risk reductions 36% (23% to 47%) and 80% (74% to 84%), respectively. Among patients who experienced delta- and alpha-variant infection, 93 (18.8%) and 65 (13.1%) had partial vaccination, while 54 (10.9%) and 29 (5.8%) had complete vaccination. Significantly weaker protective actions of partial and complete vaccination against the occurrence of delta-variant infection and illness, with respect to outcomes from the alpha-variant, were observed (Table 2).

**Table 2:** Main effects of natural (previous infection) and induced (partial or complete vaccination) exposure to SARS-CoV-2 on the relative risk reduction (RRR) of the onset of any and symptomatic infections caused by delta and alpha variants, and corresponding 95% confidence interval

	Controls	Delta cases		Alpha cases		
	*N* (%)	*N* (%)	RRR (95% CI) ^a^	*N* (%)	RRR (95% CI) ^a^	*p* value ^‡^
**Any infection (symptomatic and asymptomatic together)**	(*N* = 4960)	(*N* = 496)		(*N* = 496)		
Previous infection						
Unlike	4411 (88.9)	490 (98.8)	0% (reference)	487 (98.2)	0% (reference)	
Ascertained	549 (11.1)	6 (1.2)	90% (76% to 95%)	9 (1.8)	85% (70% to 92%)	0.547
Vaccination						
No	2650 (53.4)	349 (70.4)	0% (reference)	402 (81.1)	0% (reference)	
Partial	876 (17.7)	93 (18.8)	29% (7% to 45%)	65 (13.1)	62% (48% to 71%)	0.001
Complete	1434 (28.9)	54 (10.9)	75% (66% to 82%)	29 (5.8)	90% (85% to 94%)	0.003
**Symptomatic (mild, severe, critical and fatal together) infection**	(*N* = 3740)	(*N* = 374)		(*N* = 374)		
Previous infection						
Unlike	3332 (89.1)	371 (99.2)	0% (reference)	368 (98.4)	0% (reference)	
Ascertained	408 (10.9)	3 (0.8)	93% (79% to 98%)	6 (1.6)	87% (71% to 94%)	0.410
Vaccination						
No	1996 (53.4)	269 (71.9)	0% (reference)	312 (83.4)	0% (reference)	
Partial	697 (18.6)	71 (19.0)	35% (12% to 51%)	46 (12.3)	66% (52% to 76%)	0.005
Complete	1047 (28.0)	34 (9.1)	81% (71% to 87%)	16 (4.3)	93% (88% to 96%)	0.004

^a^ Relative risk reduction (RRR) calculated as 1- adjusted odds ratio. The latter was estimated with conditional logistic regression, adjusted for the number of previous contacts with the Regional Health Service, use of corticosteroids, drugs for chronic pain, oral anticoagulant agents and insulin, and the presence of anaemias, chronic respiratory disease, dyslipidaemia, depression, hypertension, coronary and peripheral vascular disease, hypothyroidism, epilepsy and recurrent seizures, psychosis, diabetes without insulin therapy, malignancies, other diseases of the respiratory system, other diseases of the digestive system, other diseases of the genitourinary system, gout, autoimmune disease, other diseases of the circulatory system, symptoms, signs and ill-defined conditions, diseases of the skin and subcutaneous tissues, arrhythmia, inflammatory bowel diseases, other mental disorders, heart failure, glaucoma and chronic kidney disease

^‡^ Chi-square testing the null hypothesis of between-variant homogeneity of the odds ratios

As expected by the above reported findings, direct comparison between delta (principal endpoint) and alpha (now considered as comparator) infections confirmed that partial and complete vaccination were both associated with a significantly increased risk of delta than alpha variants, being the corresponding odds ratios 1.89 (95% CI 1.30 to 2.77) and 2.71 (1.57 to 4.70), respectively, while there was no evidence that previous infection differently affected delta and alpha infection risks, being the corresponding odds ratio 0.84 (0.29 to 2.43) (data not shown).

**Fig. 2:**
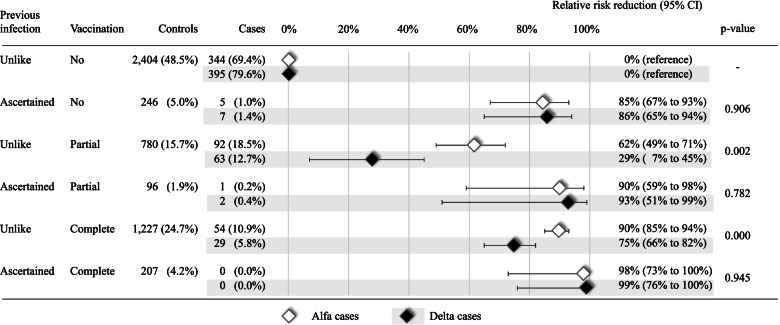
Joint effects of natural (previous infection) and induced (partial or complete vaccination) exposure to SARS-CoV-2 on the relative risk reduction (RRR) of the onset of any and symptomatic infections caused by delta and alpha variants, and corresponding 95% confidence interval. Footnote. Relative risk reduction is calculated as 1-adjusted odds ratio. The latter was estimated with conditional logistic regression, adjusted for the number of previous contacts with the Regional Health Service, use of corticosteroids, drugs for chronic pain, oral anticoagulant agents and insulin, and the presence of anaemias, chronic respiratory disease, dyslipidaemia, depression, hypertension, coronary and peripheral vascular disease, hypothyroidism, epilepsy and recurrent seizures, psychosis, diabetes without insulin therapy, malignancies, other diseases of the respiratory system, other diseases of the digestive system, other diseases of the genitourinary system, gout, autoimmune disease, other diseases of the circulatory system, symptoms, signs and ill-defined conditions, diseases of the skin and subcutaneous tissues, arrhythmia, inflammatory bowel diseases, other mental disorders, heart failure, glaucoma and chronic kidney disease. As far as citizens who had both ascertained previous infection and complete vaccine administration, as no cases of either alpha or delta infection occurred, estimates were obtained by adding a fixed value of 0.5 to the cells with zero counts

The joint action of natural and induced exposure to SARS-CoV-2 afforded protection against infection caused by the delta variant and alpha variant (Fig. 2). The weaker protection of vaccination against infection by the delta variant with respect to the alpha variant only affected individuals who had no evidence of prior infection.

### Supplementary analyses

Estimates were, in general, like those obtained in the main analyses irrespective of the time-window from non-exposure to the beneficial effects after vaccination (Additional File 1: Table S1). Among citizens who had at least one positive molecular test for a nasopharyngeal swab during the vaccination campaign, those for whom sequencing data were obtained were mainly men, of younger age, and were characterised by a lower prevalence of corticosteroid users and signs of coronary and respiratory disease with respect to those for whom sequencing data were not obtained (Additional File 1: Table S2). Finally, the rule-out approach applied for the residual unmeasured confounding analysis, showed that it is unlike that a potential confounder able to nullify the observed association exists. For example, the potential confounder should be associated to an 8-fold risk increase of infection due to delta variant, as compared to alpha variant, and, at the same time, be about 10-fold more common among vaccinated than an unvaccinated individual in order to nullify the observed association between complete vaccination and risk of infection with delta variant, as compared to alpha variant (Additional File 1: Fig. S2).

## Discussion

We showed that receipt of partial (one dose) active immunisation against COVID-19 offered lower protection against SARS-CoV-2 infection caused by the delta variant than that by the alpha variant, with a reduction of the risk of symptomatic disease of 35% and 66%, respectively. These data are in accordance with the results of a recent study showing a lower efficacy of one-dose vaccination in protecting against symptomatic disease caused by the delta variant than that by the alpha variant [10].

Our study offers at least three original cues. First, we observed that, other than symptomatic illness, between-variant differences also affected infections as a whole (symptomatic and asymptomatic) after receiving partial (one dose) active immunisation, with a corresponding relative risk reduction of 29% and 62%, respectively, for delta and alpha cases. Second, our findings showed that, although with a lower extension, between-variant differences remained even after receipt of complete (two doses) active immunisation, with a corresponding relative risk reduction of 81% and 93% for symptomatic illness, and 75% and 90% for SARS-CoV-2 infection as a whole, respectively, for delta and alpha cases. The inconsistency of the latter evidence with recent findings by Bernal et al. [10] is apparent. Their statement that “only modest differences in vaccine effectiveness were noted with the delta variant as compared with the alpha variant after the receipt of two vaccine doses” should be reconsidered in the light of our estimates comparison, being 80% and 88% the effectiveness of two vaccine doses in protecting symptomatic delta and alpha illness according to Lopez Bernal et al., 81% and 93% our corresponding findings. Finally, we observed that a previous SARS-CoV-2 infection reduces the risk of a new infection. No evidence of a different effect of a previous infection on the risk of re-infection was observed between delta and alpha variant, even if the number of individuals with a previous infection was too small for detecting a potential difference. The latter finding confirms and expands previous reports showing that (i) natural immunity appears to confer a protective effect for at least a year, which is similar to the protection reported in recent vaccine studies [11]; (ii) memory antibodies selected over time by the natural infection have greater potency and breadth than antibodies elicited by vaccination [12]. According to our findings, the joint action of the previous infection and complete active immunisation entirely avoids a new infection caused by the delta variant or alpha variant. It should be noticed, however, that the statistical power was too low to provide a precise estimate of this relative benefit. This is in line with a recent report showing that a 25- to 100-folds higher antibody response driven by memory B cells and CD4+ T cells and broader cross-protection from variants is generated when natural immunity is combined with vaccine-induced immunity [13].

The 1:1:10 matched design we used in the current study limited the number of patients included in the study, selecting only about one third of patients initially identified to be infected with the delta variant. However, it allowed to make delta and alpha cases directly comparable, thus, to directly estimate the effect of natural and induced infection to SARS-CoV-2 against a new infection due to delta variant, as compared to alpha variant.

The main strength of the present study was its population-based approach implemented in a setting with regional health services providing free access to healthcare and well-defined and near-complete follow-up based on computerised registries with full population coverage and daily updates [14]. The profiling of the target population through the clinical ‘footprints’ of real patients as they accessed their routine medical care was also a strength of our study. In addition, several sensitivity analyses confirmed the robustness of our study results.

The present study also had potential weaknesses. First, power concerns prevented investigation of the effect of the vaccine type. However, relevant between-variant differences in the differential effects of available vaccines are not expected [10], so this issue may not have affected our findings. Second, selection bias could not be excluded. Fewer than one-quarter of vaccination candidates provided a nasopharyngeal swab during the vaccination campaign, and <3% of positive nasopharyngeal swabs were sequenced for variant identification. We observed that sequenced samples belonged mainly to healthier citizens (Additional File 1: Table S2), so we suspect that differences in health-seeking behaviour depending on whether a person did or did not provide a nasopharyngeal swab that underwent sequencing may have generated a selective pressure towards the inclusion of healthier citizens. Third, misclassification of exposures and outcomes cannot be ruled out. In particular, few citizens underwent nasopharyngeal swab testing during the first wave of the COVID-19 epidemic, so some citizens may have been classified as ‘not exposed’ to natural infection despite having been infected. One may speculate that only cases with a diagnosis supported by instrumental evidence were included; the *rationale* behind this approach was that a high level of specificity of case selection is crucial to avoid misclassification, whereas sensitivity is less important [15]. Conversely, a low specificity of testing using the polymerase chain reaction could result in cases and controls being misclassified, as well as affecting one variant more than another variant, thereby resulting in attenuation of the estimates of vaccine action and affecting the between-variant comparison in a predictable direction. Finally, as with any observational study, our investigation may have been affected by differences between groups. Several attempts were made to consider such concerns, particularly by adjustment of variables. The test-negative case-control design we adopted relied on the assumption that residual confounding would affect variant-specific estimates equally, thereby making comparisons reliable [16]. The rule-out analysis suggests that it is unlikely that the observed lower vaccine protection against the delta infection with respect to the alpha one may be fully explained by an unknown and unmeasured confounder.

## Conclusions

Evidence of vaccine protection against SARS-CoV-2 infection and symptomatic COVID-19 was confirmed by our large population-based investigation. Most protection was obtained after the completion of a vaccination cycle, so efforts to maximize vaccine uptake with two doses should be boosted [17]. Lower protection towards infection and illness caused by the delta variant than by at by the alpha variant was observed, even after completion of the vaccination cycle. This finding would support efforts to maximize both vaccine uptake with two doses and fulfilment with individual protection measures [18], especially as the delta variant is rampant worldwide presently.

## Methods

### Data sources

This is a linked health administrative database study. Four population-based data sources collecting individual health information were used. The first data source was the health registry, that since the year 2000 reports and updates data on Lombardy residents, including date and causes of entry (birth, immigration) and exit (death, emigration) from the condition of the beneficiary of the Regional Health Service (RHS). The second data source was the healthcare utilisation database, which since the year 2000 collects various types of information, including inpatient diagnoses supplied by public or private hospitals, and outpatient drug and services supplied by the RHS departments. The third data source was the coronavirus disease 2019 (COVID-19) vaccination registry established on 27 December 2020 (i.e., the date of the first vaccine administration in Lombardy) which collect individual data on the date, type, and dose of vaccine dispensing. The fourth data source was the registry of patients with a confirmed diagnosis of SARS-CoV-2 infection which was established on 21 February 2020 (i.e., the date of the first ascertained diagnosis in Lombardy) for monitoring individual data on infections (ascertained through nasopharyngeal swamps and assessed from a laboratory accredited by the Regional Authority), and hospital admissions, emergency-room access, and deaths due to COVID-19. Since the initiation of the SARS-CoV-2 registry, an increasing proportion of nasopharyngeal swabs testing positive for the nucleic acids of SARS-CoV-2 have been sequenced and possibly assigned to a variant based on mutations [19]. Details on the sequencing methods used in the current study are provided in Additional file 1: Annex S1.

These administrative databases were linked through a single individual identification code. To preserve privacy, each identification code was deidentified automatically, with this inverse process being allowed only for the RHS on request from judicial authorities. Further details of the healthcare databases used in the context of COVID-19 in Lombardy have been reported [20].

### Study design

Selection of the individuals included in this case-control study started from beneficiaries of the RHS, residents in Lombardy Region, who were candidates for vaccination against COVID-19 on 27 December 2020 (i.e., when the vaccination campaign started in Italy). These include individuals who had celebrated their 12th birthday or would do so in the course of 2021. Among these, were identified individuals who during the vaccination campaign (from 27 December 2020 until 16 July 2021, i.e., the endpoint of the current study) had provided a nasopharyngeal swab for testing at least once. Among those who were positive at least once according to a molecular test, were identified those whose whole-genome sequencing was used to identify VoC .For each individual for whom the delta variant was found at a certain date (index date), one individual was found to have the alpha variant on the same date, and 10 individuals who, despite having provided a nasopharyngeal swab, were found to be negative until the index date, were selected randomly to be matched for sex and age (±1 year). By design, a case can be selected as a control for an earlier case. In this way, a 1:1:10 matching design was realised by which members of each risk set were expected to have accumulated a previous time period of the same length during which the delta case, the alpha case, and 10 controls could have experienced natural (ascertained previous infection, from 21 February 2020 to the index date) or induced (partial or complete vaccination, from 27 December 2020 to the index date) exposure to SARS-CoV-2. The corresponding design has been denoted as a “test-negative case-control design” because cases and controls were selected from candidates for vaccines who were submitted to molecular testing, and comparators (controls) were selected from those who were negative upon molecular testing [10, 16]. We expect that if exposure to SARS-CoV-2 (natural or induced) had the same strength for protecting against new infection by each variant, a similar proportion of previous exposure would be expected among cases (with either variant) and controls. Conversely, if natural exposure or induced exposure were less effective against the delta variant than against the alpha variant, then a higher proportion of previous exposure would be expected among individuals infected with the delta variant.

### Exposure to SARS-CoV-2

The most recent date of nasopharyngeal swab-positivity experienced by each case and control from 21 February 2020 to 3 months before the index date was identified from the regional SARS-CoV-2 registry [21]. From this source, cases and controls with a diagnosis of previous infection (ascertained by positivity of molecular tests of nasopharyngeal swabs) were identified on the assumption that a previous infection was less likely to have occurred in the remaining patients. Thus, previous infection was classified in two mutually exclusive categories: “ascertained”, if a nasopharyngeal swab-positivity was found, or “unlike”, otherwise.

In addition, information on the date, type, and dose of vaccine administered to each case and control were identified from the regional COVID-19 vaccination registry. Each person included was classified in three mutually exclusive categories of (i) “unexposed” (if he/she did not receive a vaccine at the endpoint date [i.e., before 16 July 2021] or if the time-window between the first or unique dose of the vaccine and the index date was <14 days); (ii) “partially vaccinated” (if he/she had received the first dose of a vaccine manufactured by Pfizer–BioNTech, Moderna, or Oxford–AstraZeneca from >14 days before the index date); (iii) “fully vaccinated” (if he/she had received the second dose of a vaccine manufactured by Pfizer–BioNTech, Moderna, or Oxford–AstraZeneca, or a unique dose of a vaccine manufactured by Janssen, from >14 days before the index date).

### Additional data

Cases and controls were classified by recording their age and sex at the index date. Hospital admissions experienced and drug prescriptions administered within 2 years before the index date were used to investigate 62 conditions that could affect the risks of severe/fatal clinical manifestations of SARS-CoV-2 infection and of experiencing adverse events [22–24]. The total number of contacts that each case and control had with the RHS within 2 years before the index date was also recorded. A complete list of these conditions, along with the International Classification of Diseases-9 and Anatomical Therapeutic Chemical codes used to track them, is provided in Additional file 1: Table S3.

### Statistical analysis

The McNemar test for paired data (or its version for the trend) was used for comparing certain measured characteristics of people infected with the delta variant and alpha variant and corresponding controls.

Conditional logistic-regression models were fitted to estimate the odds ratio and corresponding 95% confidence interval for SARS-CoV-2 infection caused by the delta variant (and separately by the alpha variant) associated with the exposure of interest. Relative risk reduction was calculated as 1-odds ratio. Models were used first to evaluate the main effects of natural and induced exposure to SARS-CoV-2. Subsequently, with the aim of evaluating their joint effect, the interaction term between natural exposure and induced exposure was included. All estimates were adjusted by including, among the additional data reported above, covariates to which ≥1% of the controls were exposed (see Table 1). Between-variant differences were tested by using a Chi-square statistic for homogeneity of odds ratios [25]. A 0.5 fixed value was added to all cells for strata where at least a cell with zero count was observed [26].

Four additional analyses were undertaken. First, only symptomatic cases with sequencing data and corresponding controls were considered. Second, due to the arbitrary nature of the choice of the time-window after vaccination during which no exposure to vaccine benefits was assumed because immunity was building gradually [14], the 14-day window used in the main analysis was shortened (7 days) and lengthened (21 days) in supplementary analyses. Third, with the aim of verifying the implications of the selection process shown in Fig. 1, the characteristics of people as they were selected for inclusion into the study population were compared. Fourth, with the aim of accounting for the potential bias associated with residual unmeasured confounders, we detected the extension of the confounding required to fully account for the exposure-outcome association, that is by using the rule-out approach described by Schneeweiss [27]. Details are reported in Additional File 1: Annex S2**.**

All analyses were performed using SAS 9.4 (Cary, NC, USA). For all hypothesis testing, *p* < 0.05 (two-tailed) was considered significant.

We used the RECORD (REporting of studies Conducted using Observational Routinely-collected health Data) checklist when writing our manuscript [28].

## Supplementary Information

**Additional file 1:** Trend of new infections sustained by delta and alpha variants observed between May and July 2021 (**Fig. S1**); Influence of a potential unmeasured confounder on the relationship between complete vaccination (exposure) and risk of infection due to delta variant, as compared to alpha variant (outcome) (**Fig. S2**); Main effects of partial and complete vaccination on the onset of new infections caused by delta and alpha variants. Time-windows of 7 and 21 days from vaccine inoculation until immune response (alternative to 14 days of the main analysis), were assumed (**Table S1**); Comparing selected characteristics of citizens who had at least a positive molecular test of nasopharyngeal swab during the vaccination campaign according whether the corresponding whole-genome sequencing was obtained or less (**Table S2**); List of conditions used for typifying the study populations (**Table S3**); SARS-CoV-2 testing and variant identification methods used by the laboratories accredited from Health authorities of Lombardy Region (**Annex S1**); Details of the rule-out approach applied for the residual unmeasured confounding analysis (**Annex S2**).

## Data Availability

The data that support the findings of this study are available from Lombardy Region, but restrictions apply to the availability of these data, which were used under license for the current study, and so are not publicly available. Data are however available from the Lombardy Region upon reasonable request.

## Abbreviations

CI: Confidence interval
COVID-19: Coronavirus disease 2019
RHS: Regional Health Service
SARS-CoV: Severe acute respiratory syndrome-coronavirus
VoC: Variants of Concern
