# Supplementary material for: Protective action of natural and induced immunization against the occurrence of delta or alpha variants of SARS-CoV-2 infection: a test-negative case-control study
Source: BMC Med. 2022 Feb 8;20:52. doi: 10.1186/s12916-022-02262-y (PMC8821854; doi:10.1186/s12916-022-02262-y)
Supplement: Supplementary file 1 — Additional file 1: Trend of new infections sustained by delta and alpha variants observed between May and July 2021 (Fig. S1); Influence of a potential unmeasured confounder on the relationship between complete vaccination (exposure) and risk of infection due to delta variant, as compared to alpha variant (outcome) (Fig. S2); Main effects of partial and complete vaccination on the onset of new infections caused by delta and alpha variants. Time-windows of 7 and 21 days from vaccine inoculation until immune response (alternative to 14 days of the main analysis), were assumed (Table S1); Comparing selected characteristics of citizens who had at least a positive molecular test of nasopharyngeal swab during the vaccination campaign according whether the corresponding whole-genome sequencing was obtained or less (Table S2); List of conditions used for typifying the study populations (Table S3); SARS-CoV-2 testing and variant identification methods used by the laboratories accredited from Health authorities of Lombardy Region (Annex S1); Details of the rule-out approach applied for the residual unmeasured confounding analysis (Annex S2). [file 12916_2022_2262_MOESM1_ESM.docx]

**Additional file 1**

**Figure S1**. Trend of new infections sustained by delta and alpha variants observed between May and July 2021.


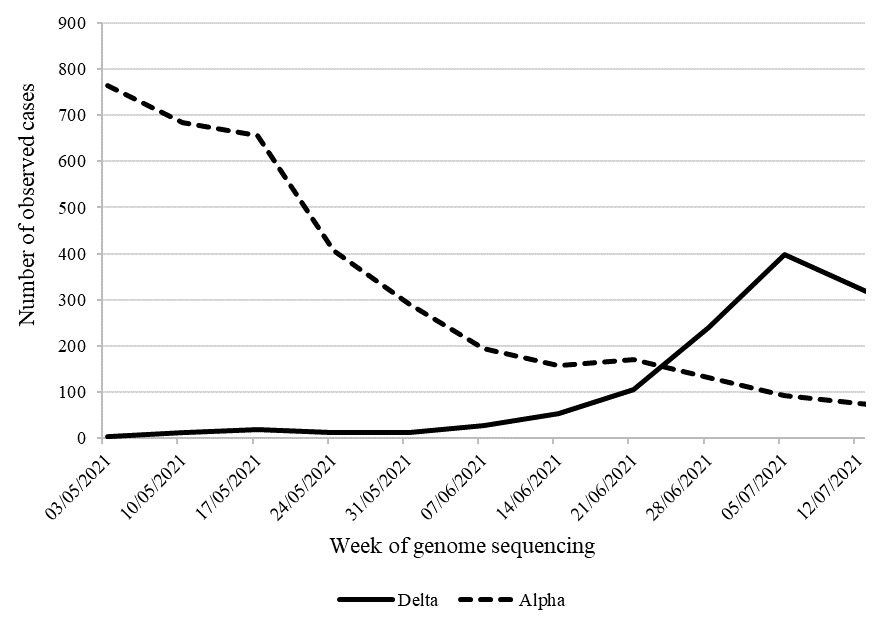


**Figure S2**. Influence of a potential unmeasured confounder on the relationship between complete vaccination (exposure) and risk of infection due to delta variant, as compared to alpha variant (outcome).


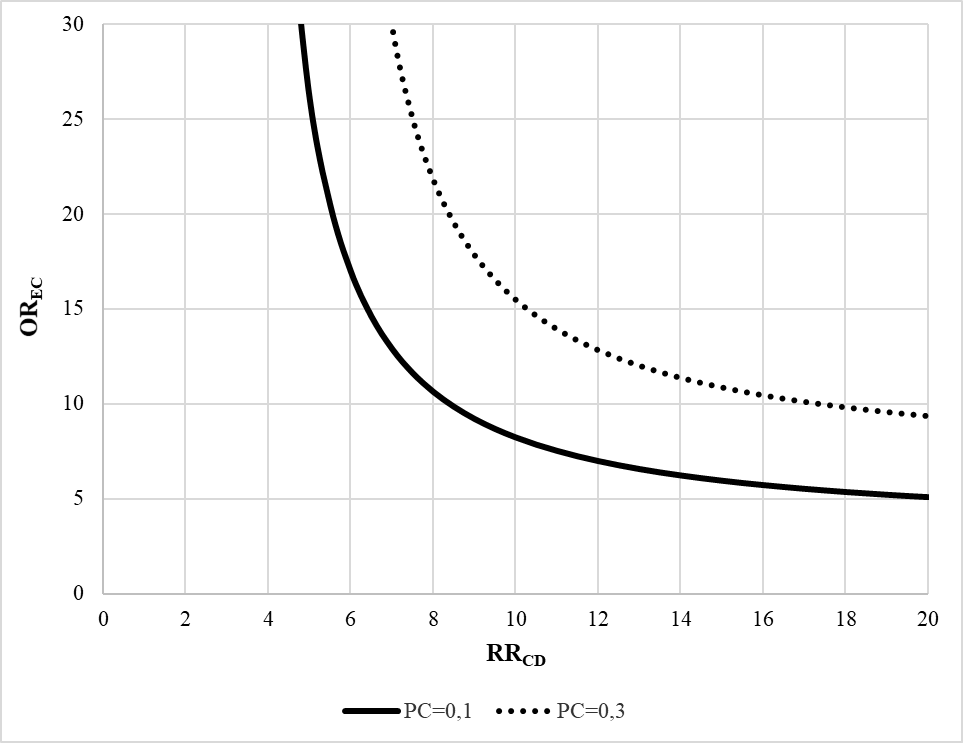


Footnote: The graph indicates the RR_CD_-OR_CE_ combinations (that is, the confounder-outcome and the confounder-exposure associations, respectively) that would be required to move the observed association (that is, OR=2.71) towards the null. Let assume the unmeasured confounder to have a prevalence of 10% in the study population. This confounder, for example, should be associated to an 8-fold risk increase of infection due to delta variant (RR_CD_=8) and, at the same time, be about 10-fold more common among vaccinated than unvaccinated individual (OR_EC_=10) in order to nullify the observed association. If the prevalence of the unmeasured confounder were 30%, then this confounder should be even more strongly associated with both the outcome and the exposure (e.g., it should be associated to an 8-fold risk increase of infection due to delta variant (RR_CD_=8) and, at the same time, be about 20-fold more common among vaccinated than unvaccinated individual (OR_EC_=10) in order to nullify the observed association).

**Table S1**. Main effects of partial and complete vaccination on the onset of new infections caused by delta and alfa variants. Time-windows of 7 and 21 days from vaccine inoculation until immune response (alternative to 14 days of the main analysis), were assumed

**7 days**

|  | Controls | Delta cases | | Alfa cases | | p-value‡ |
| --- | --- | --- | --- | --- | --- | --- |
| Vaccination | N (%) | N (%) | OR (95% CI)† | N (%) | OR (95% CI)† |  |
| No | 2,247 (49.3) | 310 (62.5) | 1.00 (reference) | 365 (73.6) | 1.00 (reference) |  |
| Partial | 985 (19.9) | 130 (26.2) | 0.99 (0.78 to 1.25) | 98 (19.8) | 0.58 (0.45 to 0.75) | 0.003 |
| Complete | 1,528 (30.8) | 56 (11.3) | 0.26 (0.19 to 0.35) | 33 (6.6) | 0.11 (0.08 to 0.17) | <0.001 |

**21 days**

|  | Controls | Delta cases | | Alfa cases | | p-value‡ |
| --- | --- | --- | --- | --- | --- | --- |
| Vaccination | N (%) | N (%) | OR (95% CI) † | N (%) | OR (95% CI) † |  |
| No | 2,843 (57.3) | 372 (75.0) | 1.00 (reference) | 417 (84.1) | 1.00 (reference) |  |
| Partial | 768 (15.5) | 74 (14.9) | 0.62 (0.47 to 0.83) | 53 (10.7) | 0.35 (0.25 to 0.48) | 0.010 |
| Complete | 1,349 (27.2) | 50 (10.1) | 0.25 (0.18to 0.34) | 26 (5.2) | 0.10 (0.06 to 0.15) | 0.003 |

† Adjusted odds ratio, and 95% confidence interval, estimated with conditional logistic regression, adjusted for the number of previous contacts with the Regional Health Service, use of corticosteroids, drugs for chronic pain, oral anticoagulant agents and insulin, and the presence of anaemias, chronic respiratory disease, dyslipidaemia, depression, hypertension, coronary and peripheral vascular disease, hypothyroidism, epilepsy and recurrent seizures, psychosis, diabetes without insulin therapy, malignancies, other diseases of the respiratory system, other diseases of the digestive system, other diseases of the genitourinary system, gout, autoimmune disease, other diseases of the circulatory system, symptoms, signs and ill-defined conditions, diseases of the skin and subcutaneous tissues, arrhythmia, inflammatory bowel diseases, other mental disorders, heart failure, glaucoma and chronic kidney disease.

‡ Chi-square testing the null hypothesis of between-variant homogeneity of the exposure effect

**Table S2**. Comparing selected characteristics of citizens who had at least a positive molecular test of nasopharyngeal swab during the vaccination campaign according whether the corresponding whole-genome sequencing was obtained or less

|  | Sequenced N = 11,379 | Not sequenced N = 408,035 | Standardized mean difference† |
| --- | --- | --- | --- |
|  |  |  |  |
| Men | 6,061 (53.3%) | 200,732 (49.2%) | +0.11 |
| Age category |  |  |  |
| 12 to 49 yr | 6,430 (56.5%) | 200,516 (49.1%) | +0.17 |
| 50 to 59 yr | 1,751 (15.4%) | 83,519 (20.5%) | -0.12 |
| 60 to 69 yr | 1,154 (10.1%) | 52,467 (12.9%) | -0.07 |
| 70 to 79 yr | 963 (8.5%) | 35,463 (8.7%) | -0.00 |
| ≥ 80 yr | 1,081 (9.5%) | 36,070 (8.8%) | +0.03 |
| Number of previous contacts with NHS ^‡^ |  |  |  |
| < 5 | 6,027 (53.0%) | 208,815 (51.2%) | +0.06 |
| 5 to 99 | 4,314 (37.9%) | 169,780 (41.6%) | -0.05 |
| ≥ 100 | 1,038 (9.1%) | 29,440 (7.2%) | +0.08 |
| Users of selected drugs ^‡§^ |  |  |  |
| Corticosteroids | 1,194 (10.5%) | 71,529 (17.5%) | -0.19 |
| Drugs for chronic pain | 327 (2.9%) | 10,409 (2.6%) | +0.02 |
| Oral anticoagulant agents | 405 (3.6%) | 13,354 (3.3%) | +0.02 |
| Insulin | 537 (4.7%) | 17,463 (4.3%) | +0.02 |
| Patients suffering selected diseases ^‡§^ |  |  |  |
| Anaemias | 977 (8.6%) | 34,483 (8.5%) | +0.01 |
| Chronic respiratory disease | 1,272 (11.2%) | 43,569 (10.7%) | +0.03 |
| Dyslipidaemia | 1,374 (12.1%) | 49,838 (12.2%) | -0.01 |
| Depression | 841 (7.4%) | 32,808 (8.0%) | -0.02 |
| Hypertension | 1,255 (11.0%) | 47,777 (11.7%) | -0.01 |
| Coronary and peripheral vascular disease | 766 (6.7%) | 55,722 (13.7%) | -0.22 |
| Hypothyroidism | 468 (4.1%) | 17,119 (4.2%) | -0.00 |
| Epilepsy and recurrent seizures | 193 (1.7%) | 6,791 (1.7%) | -0.01 |
| Psychosis | 270 (2.4%) | 9,441 (2.3%) | +0.01 |
| Diabetes without insulin therapy | 537 (4.7%) | 17,463 (4.3%) | +0.03 |
| Malignancies | 261 (2.3%) | 8,659 (2.1%) | +0.02 |
| Other diseases of the respiratory system | 229 (2.0%) | 35,885 (8.8%) | -0.30 |
| Other diseases of the digestive system | 226 (2.0%) | 9,548 (2.3%) | -0.02 |
| Other diseases of the genitourinary system | 199 (1.7%) | 9,477 (2.3%) | -0.04 |
| Gout | 390 (3.4%) | 11,674 (2.9%) | +0.04 |
| Autoimmune disease | 220 (1.9%) | 8,312 (2.0%) | -0.00 |
| Other diseases of the circulatory system | 247 (2.2%) | 9,665 (2.4%) | -0.01 |
| Symptoms, signs and ill-defined conditions | 150 (1.3%) | 6,652 (1.6%) | -0.02 |
| Diseases of the skin and subcutaneous tissues | 134 (1.2%) | 5,453 (1.3%) | -0.01 |
| Arrhythmia | 278 (2.4%) | 10,186 (2.5%) | -0.00 |
| Inflammatory bowel diseases | 159 (1.4%) | 5,903 (1.4%) | +0.00 |
| Other mental disorders | 38 (0.3%) | 1,623 (0.4%) | -0.01 |
| Heart failure | 234 (2.1%) | 7,491 (1.8%) | +0.02 |
| Glaucoma | 203 (1.8%) | 6,755 (1.7%) | +0.01 |
| Chronic kidney disease | 79 (0.7%) | 2,047 (0.5%) | +0.03 |
| Severity of Covid-19 symptoms ^#^ |  |  |  |
| Asymptomatic | 6,463 (56.8%) | 277,921 (68.1%) | -0.24 |
| Mild | 1,940 (17.0%) | 77,082 (18.9%) | -0.05 |
| Severe | 2,213 (19.4%) | 42,903 (10.5%) | +0.25 |
| Critical | 314 (2.8%) | 2,936 (0.7%) | +0.16 |
| Fatal | 449 (3.9%) | 7,193 (1.8%) | +0.13 |

† Due to the large sample size of the study groups, we avoided comparisons based on statistical tests, and the standardized mean differences were then calculated. Standardized mean differences < 0.10 should be considered negligible

**Table S3.** List of conditions used for typifying the study populations

| **Diagnostic categories** | **#** | **Disease / condition** | **ICD-9 CM** | **ATC** |
| --- | --- | --- | --- | --- |
| Infectious and parasitic diseases | 1 | HIV infection | 042.x, V08 | J05AB14, J05AE, J05AF01, J05AF02, J05AF04, J05AF05, J05AF06, J05AF09. J05AG, J05AR, J05AX07, J05AX08, J05AX09, J05AX12 |
|  | 2 | Tuberculosis and Other infectious and parasitic diseases | 010.x - 018.x, 001.x-009.x, 020.x-027.x, 030.x-0.41.x, 045.x–057.x, 060.x-066.x, 070.x-088.x, 090.x-104.x, 110.x-118.x, 120.x-139.x | J04AB |
| Neoplasms | 3 | Solid malignancies and Neoplasm of lymphatic and haematopoietic tissue | 140.x-165.x, 170.x-176.x, 179.x-199.x, V58.0, 92.2, 200.x-208-x | L01, L03AC, L02BA01, L02BA02, L02BG02, L02BG03, L02BG04, L02BG06, L02BB01, L02BB03, L02AE02, L02AE04, L02AB01 |
|  | 4 | Benign neoplasm and carcinoma in situ | 210.x-234.x |  |
| Endocrine, nutritional and metabolic diseases, and immunity disorders | 5 | Hypothyroidism | 243, 244.x | H03A, H03B |
|  | 6 | Hyper e hypoparathyroidism | 252.0, 252.1 |  |
|  | 7 | Diabetes without insulin therapy | 250.x, 348.0x, 357.2, 362.0, 366.41 | A10B |
|  | 8 | Insulin therapy |  | A10A |
|  | 9 | Dyslipidaemia | 272.2, 272.4 | C10 |
|  | 10 | Obesity | 278.0x |  |
|  | 11 | Weight loss | 260-263.x |  |
|  | 12 | Disorders of fluid, electrolyte, and acid-base balance | 276.x |  |
|  | 13 | Gout | 274.x | M04AC01, M04AA, M04AB |
|  | 14 | Other disorders of endocrine, nutritional and metabolic diseases | 240.x-242.x, 245.x, 246.x, 249.x, 251.x, 252.8, 252.9, 253.x-259.x, 264.x-269.x, 270.x, 271.x, 272.0, 272.1, 272.3, 272.5-272.9, 273.x, 275.x, 277.x, 278.1-278.8 (except 277.0) |  |
|  | 15 | Disorders involving the immune mechanisms | 279.x |  |
| Diseases of the blood and blood-forming organs | 16 | Coagulation defects | 286.x | B02B |
|  | 17 | Autoimmune haemolytic anaemias, Other anaemias, Anaemias only tracked from drug therapy | 280.x-282.x, 283.1-283.9, 284.x-285.x | B03A, B03B, B03XA01, L03AA |
|  | 18 | Other diseases of the blood and blood-forming organs | 287.x-289.x |  |
| Mental disorders | 19 | Dementia / Alzheimer | 290.0-290.4x, 331.0x | N06DA, N06DX01 |
|  | 20 | Psychosis | 295.x, 297.x, 298.2-298.9, 299.1x | N05AD, N05AA, N05AB, N05AC, N05AX, N05AE, N05AF, N05AG N05AH, N05AL |
|  | 21 | Depression | 296.2, 296.3, 296.82, 298.0, 300.4, 301.12, 309.0x, 309.1x, 311.x | N06A |
|  | 22 | Bipolar disorders | 296.0x, 296.1x, 296.4x, 296.5x, 296.6x, 296.7x, 296.80, 296.81, 296.89, 296.9x, 298.1x | N05AN |
|  | 23 | Alcohol abuse | 291.1, 291.2, 291.5, 291.8x, 291.9, 303.9, 305.0x, V11.3x | N07BB01 |
|  | 24 | Drug addition | 292.0x, 292.82-292.89, 292.9x, 304.x, 305.2x-305.9x | N07BB04 |
|  | 25 | Anxiety | 300.0x | N05BA, N05BB01, N05CD, N05BC01, N05BC51, N05BX, N05CF, N05CX01, N06BX |
|  | 26 | Other mental disorders | 290.8, 290.9, 291.0, 291.3, 291.4, 292.1x, 292.2, 292.81, 293.x, 294.x, 299.0x, 299.8x, 299.9x, 300.0x-300.2x, 300.3, 300.5-300.9, 301.0, 301.10, 301.11, 301.2x-301.9x, 302.x, 303.x, 305.1, 306.x-308.x, 309.2x-309.4x, 310.x, 312.x-319.x |  |
| Diseases of the nervous system and sense organs | 27 | Parkinson’s disease and parkinsonism | 332.x | N04 |
|  | 28 | Multiple sclerosis | 340 | L03AB07, L03AB08, L04AA23, L04AA27, L03AX13, L04AA31, L04AA34, L03AB13, L04AX07 |
|  | 29 | Epilepsy and recurrent seizures | 345.x | N03AF01, N03AB02, N03AA02, N03AA03, N03AA04, N03AE01, N03AD01, N03AG01, N05BA09, N03AG04, N03AX10, N03AG06, N03AF02, N03AX14, N03AX15 |
|  | 30 | Glaucoma | 365.x | S01E |
|  | 31 | Disorders of the eye and adnexa | 360.x-379.x (except 365.x) |  |
|  | 32 | Diseases of the ear and mastoid process | 380.x-389.x |  |
|  | 33 | Other diseases of the nervous system and sense organs | 320.x-326.x, 330.x-331.x, 333.x-337.x, 340.x-344.x, 346.x-359.x |  |
| Diseases of the circulatory system | 34 | Ischaemic Heart Disease/Angina | 410.x – 414 | C01DA, C01DX |
|  | 35 | Heart failure | 398.91, 402.11, 402.91, 404.11, 404.13, 404.91, 404.93, 428.x |  |
|  | 36 | Arrhythmia | 426.10, 426.11, 426.13, 426.20-426.53, 426.60-426.89, 427.0, 427.2, 427.31, 427.60,427.9, 785.0x, V45.0x, V53.3x | C01BA, C01BC, C01BD |
|  | 37 | Valvular diseases | 093.20-093.24, 394.0x-397.1x, 424.00-424.91, 746.3x-746.6x, V42.2x, V43.3x |  |
|  | 38 | Vascular diseases | 440.x, 441.2, 441.4, 441.7, 441.9, 443.1x-443.9x, 447.1, 557.1x, 557.9x, 785.4x, V43.4x |  |
|  | 39 | Cerebrovascular diseases | 430.x-438.x |  |
|  | 40 | Hypertension | 401.x-405.x | C03AA, C03AB, C03AH, C03AX01, C02CA04, C03BA02, C03BA03, C03BA04, C03BA05, C03BA07, C03BA08, C03BA09, C03BA10, C03BA11, C03DB01, C03DB02, C03EA, C09BA02, C09BA03, C09BA04, C09BA05, C09BA06, C09BA07, C09BA08, C09BA09, C09BB, C09DB, C09DA01, C09DA02, C09DA03, C09DA04, C09DA06, C09DA07, C09DA08, C02AB01, C02AB02, C02AC01, C02AC02, C02AC04, C02AC05, C02DB02, C02DB03, C02DB04, C02DC01, C02DD01, C02DG01, C02KA01, C02KB01, C02KC01, C02KD01, C02KX01, C09XA |
|  | 41 | Coronary and peripheral vascular disease |  | B01AB, B01AX01, B01AD10, B01AD12, C04AD03, B01AC05 |
|  | 42 | Oral anticoagulant agents |  | B01AA, B01AE, B01AF |
|  | 43 | Other diseases of the circulatory system | 390.x-392.x, 393, 397.9, 398.90, 398.99, 411.8x, 412.x-417x, 420.x-423.x, 424.99, 425.x, 426.0, 426.12, 426.54, 426.9, 427.1, 427.32, 427.4x, 427.5, 427.61, 427.69, 427.8x, 429.x, 441.0x, 441.1, 441.3, 441.5, 441.6, 442.x, 443.0, 444.x-446.x, 447.0, 447.2-447.9, 448.x 451.x-459.x |  |
| Diseases of the respiratory system | 44 | Chronic Obstructive Pulmonary Disease, Asthma, Chronic respiratory disease only tracked from drug therapy | 490-492.x, 493.x, 494.x, 496 | R03AA, R03AB, R03AC, R03DA, R03DB, R03DA20, R01AC01, R03BC01, R01AC51, S01GX01, S01GX51, R03BA |
|  | 45 | Acute respiratory infections | 460-466.x |  |
|  | 46 | Cystic Fibrosis | 277.0 | R05CB, R05FB01, R05FA01, A09AA02, R07AX02, R07AX30, R07AX31 |
|  | 47 | Other diseases of the respiratory system | 470.x-478.x, 480.x-487.x, 495.x, 500.x-508.x, 510.x-519.x |  |
| Diseases of the digestive system | 48 | Liver cirrhosis and other liver chronic diseases | 571.x, 573.x | J05AP08, J05AP09, J05AP51, J05AP53, J05AP54, J05AP55, J05AP56, J05AP57, B05AA01 |
|  | 49 | Inflammatory bowel diseases (Ulcerative colitis and Chron’s disease) | 555.x-556.x | A07EC01, A07EC02, A07EC03, A07EC04 |
|  | 50 | Chronic and acute pancreatitis | 577.0-577.1 |  |
|  | 51 | Other diseases of the digestive system | 520.x-553.x, 557.x-570, 572.x, 574.x-576.x, 577.2-577.9, 578.x, 579.x |  |
| Diseases of the genitourinary system | 52 | Chronic kidney disease | 585, V45.1, V56.x, V03AE  580.x-584.x, 586, 587, 588.x-589.x |  |
|  | 53 | Other kidney disorders |  |  |
|  | 54 | Other diseases of the genitourinary system | 590.x-608.x, 610.x, 611.x, 614.x-629.x |  |
| Diseases of the skin and subcutaneous tissues | 55 | Diseases of the skin and subcutaneous tissues, including No rheumatoid psoriasis | 680.x-686.x, 690.x-695.x, 696.0, 696.2-696.5, 696.8, 697.x, 698.x, 700.x-709.x, 696.1 | D05BB01, D05BB02, D05AX |
| Diseases of the musculoskeletal system and connective tissue | 56 | Autoimmune disease (Rheumatoid arthritis, Rheumatoid psoriasis, Anchylosing spondylitis, Systemic sclerosis, Systemic lupus erythematosus) | 714.0, 696.0, 720.0, 710.1x, 710.0x |  |
|  | 57 | Other diseases of the musculoskeletal system and connective tissue | 710.2-710.9, 711.x-713.x, 714.1x, 714.9x, 715.x-719.x, 720.1x-720.9x, 721.x-739.x |  |
| Symptoms, signs and ill-defined conditions | 58 | Symptoms, signs and ill-defined conditions | 780-799 |  |
| Other conditions | 59 | Transplantation | V42 | L04AA01, L04AA02, L04AA03, L04AA04, L04AA05, L04AA06, L04AA08, L04AA09, L04AA10, L04AA11, L04AA12, L04AA14, L04AA15, L04AA16, L04AA17, L04AA18, L04AA19, L04AA21, L04AD01, L04AD02, L04AX01 |
|  | 60 | Chronic pain | 338.2, 338.4 | N02AA01, N02AG01, N02AE01, N02AB03, N02AA05, N02AA55, N02AA03, N02AX06 |
|  | 61 | Corticosteroids |  | H02 |
|  | 62 | Hormone therapy (oral contraceptives or replacement hormone therapy) |  | G03AA, G03AB, G02BB01, G03HB01, G03CA |

**Annex S1.** SARS-CoV-2 testing and variant identification methods used by the laboratories accredited from Health authorities of Lombardy Region

Cases were determined by using real-time reverse transcription–polymerase chain reaction (RT-PCR) testing on nasopharyngeal swabs. RT-PCR testing was performed by both private and public laboratories that are part of the NHS, which is organized under the Ministry of Health and is administered on a regional basis.

Nasopharyngeal swab genotyping was performed since the end of December 2020 in the following cases: already vaccinated subjects, reinfections, high-risk contexts (e.g., immunosuppressed), people coming from high-incidence countries, national areas with evidence of local clusters. From the beginning of May, in addition to the previous cases, all positive subjects age <19 years were also genotyped. From mid-June the overall positive cases were sequenced.

The search for variants was carried out through a first RT-PCR screening for the alpha variant, in the event of a negative outcome at the screening; sequencing was carried out with the Sanger or NGS method. The search for variants was carried out by 18 laboratories of the Lombard genotyping / sequencing network.

**Annex S2.** Details of the rule-out approach applied for the residual unmeasured confounding analysis.

With the aim of accounting for the potential bias associated with residual unmeasured confounders, we detected the extension of the confounding required to fully account for the exposure-outcome association, that is by using the “rule-out” approach described by Schneeweiss [Schneeweiss S. Pharmacoepidemiol Drug Saf. 2006;15:291-303]. Let consider the observed association between complete vaccination and the risk of infection sustained by the delta variant, as compared to the alpha variant (i.e., OR=2.71). With such approach, we estimated the extent of the association between a potential confounder and both i) the risk of infection due to delta variant, as compared to alpha variant (RR_CD_) and ii) the prevalence of the confounder among vaccinated and unvaccinated patients (OR_EC_) that would nullify the observed association (i.e. that would lead the observed association toward the null). We set the potential unmeasured confounder to: (1) have a prevalence in the study population of 10% or 30%; (2) increase the risk of infection due to delta variant, as compared to alpha variant, in patients exposed than in those unexposed to the confounder (RR_CD_>1); (3) be more common among vaccinated than unvaccinated individuals (OR_EC_>1).
